# MBE Growth of AlN Nanowires on Si Substrates by Aluminizing Nucleation

**DOI:** 10.1186/s11671-015-1083-0

**Published:** 2015-10-05

**Authors:** Yanxiong E, Zhibiao Hao, Jiadong Yu, Chao Wu, Runze Liu, Lai Wang, Bing Xiong, Jian Wang, Yanjun Han, Changzheng Sun, Yi Luo

**Affiliations:** Tsinghua National Laboratory for Information Science and Technology, Department of Electronic Engineering, Tsinghua University, Beijing, 100084 People’s Republic of China

**Keywords:** AlN nanowires, Nucleation, Crystal polarity, Molecular beam epitaxy

## Abstract

By introducing an aluminization process to achieve nucleation of nanowires (NWs), spontaneous growth of AlN NWs on Si substrates has been realized by plasma-assisted molecular beam epitaxy. The AlN NWs are grown from the nuclei formed by the aluminization process, and the NW density and diameter can be controlled by the aluminization parameters. The influence of growth conditions on the morphologies of AlN NWs is carefully investigated. Island-like films are found to grow between the NWs due to poor migration ability of Al adatoms. The films are proved to be Al-polar different from the N-polar AlN NWs, which can explain the absence of newly formed NWs. Increasing the V/III ratio can efficiently suppress the growth of Al-polar AlN films.

## Background

AlN nanowires (NWs) on Si substrates appear to be free of crystalline defects due to their free surfaces which relax the elastic strain introduced by lattice mismatch. Associated with the large bandgap as well as the chemical and physical stability, AlN NWs have advantages in many device applications such as deep ultraviolet lasers [1], photovoltaic devices [2], and optical sensors [3]. Furthermore, by introducing AlGaN heterostructures, AlGaN/AlN quantum wells or quantum dots in NWs can achieve high internal quantum efficiency even at room temperature, leading to the significant advances in single photon sources [4], electrically pumped nanolasers [5], and deep ultraviolet LEDs [6].

Unfortunately, the poor migration ability of Al adatoms brings much difficulties in the AlN nucleation and subsequent growth of AlN NWs. Additionally, the domain coincidence matching between AlN (0001) and Si (111) lattices is another obstacle for the three-dimensional growth of AlN NWs [7]. Daudin et al. [8] successfully grew AlN NWs on Si (001) by introducing an amorphous SiO_2_ layer as a buffer layer to improve the AlN NW nucleation. However, they found that, by this method, the AlN NWs suffered from severe coalescence and inevitable oxygen impurities from SiO_2_ decomposition which also had influence on the morphologies of the AlN NWs. Mi et al*.* [9] realized AlN NW growth using a GaN NW template and investigated their optical properties. It was found that the GaN NWs partially decomposed at the high growth temperature required for the growth of AlN NWs. Apart from this work, there are fairly few reports on the growth of AlN NWs, and the growth mechanism of AlN NWs is far from being fully understood.

In this work, by introducing an aluminization process [10] to complete AlN NW nucleation, the spontaneous growth of AlN NWs on Si (111) substrates is realized. This simple in situ process can avoid the impacts from templates and the incorporation of impurities except Si. The influence of growth conditions on the properties of AlN NWs is carefully investigated, and AlN NWs with improved morphologies have been obtained by optimizing the growth conditions.

## Methods

All the AlN NW samples were grown on 2-in. Si (111) substrates by a plasma-assisted molecular beam epitaxy (PA-MBE) system. Prior to being loaded into the MBE chamber, the substrates were treated by a standard RCA cleaning process. The substrates were firstly outgassed at 280 °C in the preparation chamber, then thermally cleaned at 940 °C for 15 min in the growth chamber. Before the growth, nitridation was performed at 900 °C for 60 s to obtain β-Si_3_N_4_ with an “8 × 8” reflection high-energy electron diffraction (RHEED) pattern [11]. After nitridation, the N plasma source was turned off, and the aluminization process was performed at a substrate temperature in the range of 850~950 °C for 1000 s; after that, the AlN NWs were directly grown at a substrate temperature in the range of 940~980 °C. Various Al source temperatures between 1180 and 1240 °C and N_2_ flow rates between 1.7 and 3.0 sccm were adopted to achieve different V/III ratios, while the radio frequency (RF) power of the N source was fixed at 375 W. The samples grown under different conditions are listed in Table 1. The growth procedures were monitored by RHEED and a reflectivity measurement system. Atom force microscopy (AFM) and scanning electron microscopy (SEM) were employed to analyze the surface morphologies of the samples. High-resolution transmission electron microscopy (HRTEM) was used to analyze the crystalline quality. The TEM samples were prepared by focused ion beam (FIB) slicing with a slice thickness of 100~150 nm.

**Table 1:** Growth conditions of different AlN NW samples

Samples	Aluminization temperature (°C)	Al source temperature (°C)	N_2_ flow rate (sccm)	Growth temperature (°C)	Growth time (h)
A	N.A.	1220	1.7	960	3
B	850	1220	1.7	960	1
C	900	1220	1.7	960	1
D	950	1220	1.7	960	1
E	900	1220	1.7	940	1
F	900	1220	1.7	980	1
G	900	1200	1.7	960	1
H	900	1240	1.7	960	1
J	900	1240	1.7	960	3
K	900	1220	1.7	960	3
L	900	1180	1.7	960	3
M	900	1220	2.5	960	3
O	900	1220	3.0	960	3
P	900	1240	2.5	960	3
Q	950	1240	2.5	960	3

## Results and Discussion

**Fig. 1:**
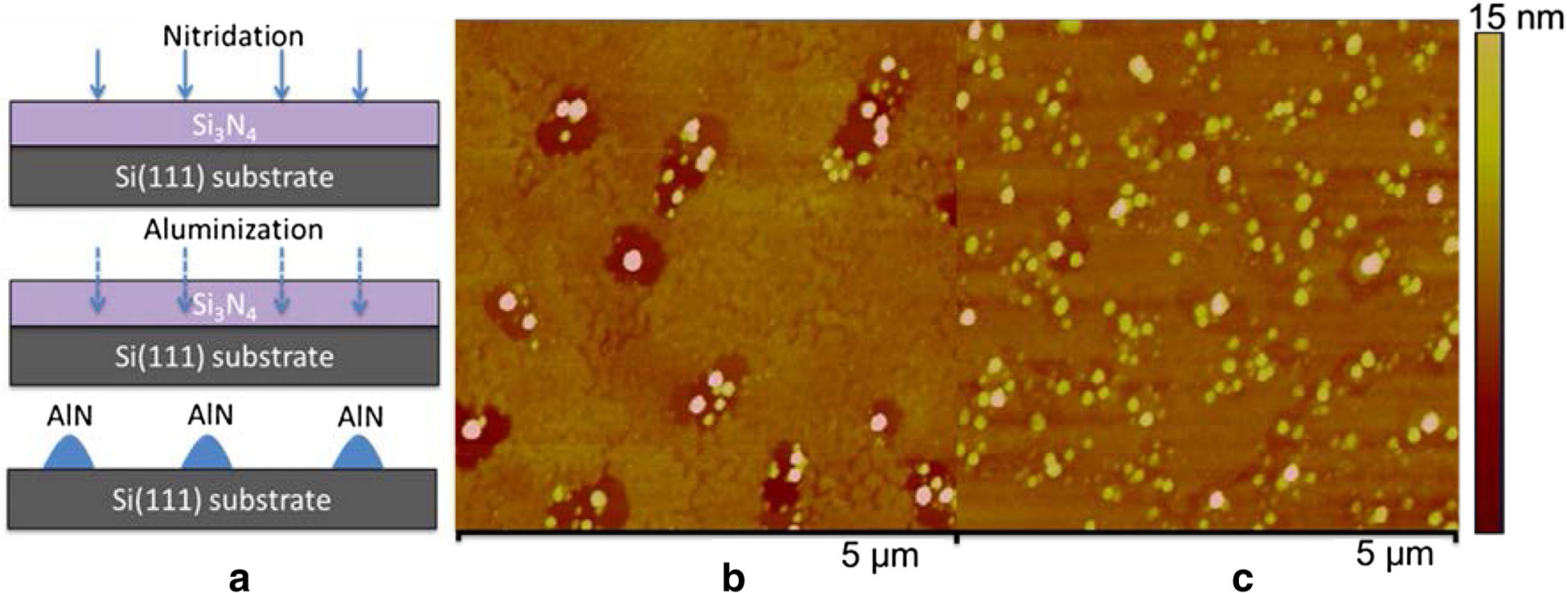
**a** A schematic illustration of the aluminization process. Al flux reacts with β-Si_3_N_4_ to form AlN islands as nuclei for AlN NW growth. AFM images of the substrates aluminized at (**b**) 850 °C and (**c**) 950 °C

A schematic illustration of the aluminization process is shown in Fig. 1a. After the nitridation process, an Al flux was irradiated for a long enough time, reacting with β-Si_3_N_4_ at high temperature to form AlN islands as nuclei for AlN NW growth. Samples right after the aluminization process were taken out of the MBE chamber and examined by AFM. Figure 1b, c shows the AFM images of the samples aluminized at 850 and 950 °C, respectively. It can be seen that the sample aluminized at higher temperature presents more uniform crystal islands with higher density and smaller average diameter. The densities of the islands formed at 850 and 950 °C are 2.4 × 10^8^ and 1.6 × 10^9^ cm^−2^, respectively.

**Fig. 2:**
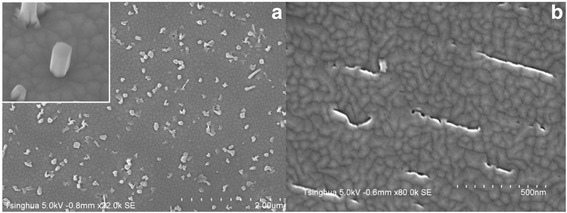
**a** SEM image of a typical AlN NW sample. Shown in the *inset* is the magnified image of a single NW. **b** SEM image of the sample without aluminizing nucleation

**Fig. 3:**
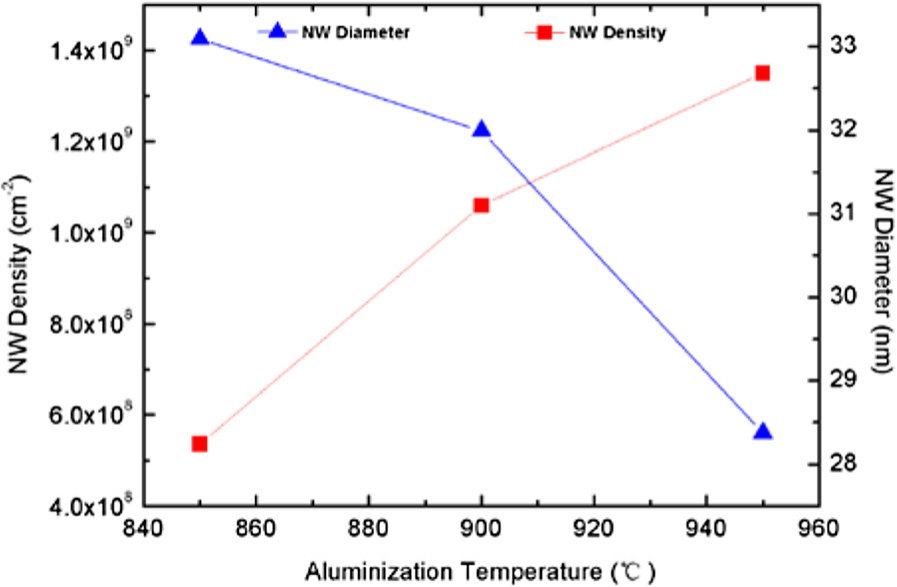
The density and average diameter of AlN NWs versus the aluminization temperature. *Solid lines* are guides to the eye

AlN NW samples B, C, and D aluminized at 850, 900, and 950 °C, respectively, were prepared to analyze the influence of the nucleation layer on the morphologies of AlN NWs. Fig. 2a is the typical SEM image of the AlN NWs, and the inset clearly shows the characteristic of a single NW. The densities and average diameters of the AlN NWs versus aluminization temperature are plotted in Fig. 3. Taking into consideration the run-to-run deviation, the densities of the AlN NWs are quite close to those of the nucleation islands of corresponding aluminization samples. It can be found that, compared to Fig. 1b, c, the sample aluminized at higher temperature has more nucleation islands with smaller average diameter, hence leading to the increase of AlN NW density and decrease of the average diameter with aluminization temperature. This implies that the AlN NWs are transformed and grown from the nucleation islands. As a contrast, sample A without aluminization process was grown under the same conditions as sample C for an elongated growth time of 3 h. However, as witnessed by the SEM image in Fig. 2b, there is an island-like film grown on the substrate instead of NWs. Therefore, it can be concluded that the aluminization process can efficiently improve the nucleation of AlN NWs.

**Fig. 4:**
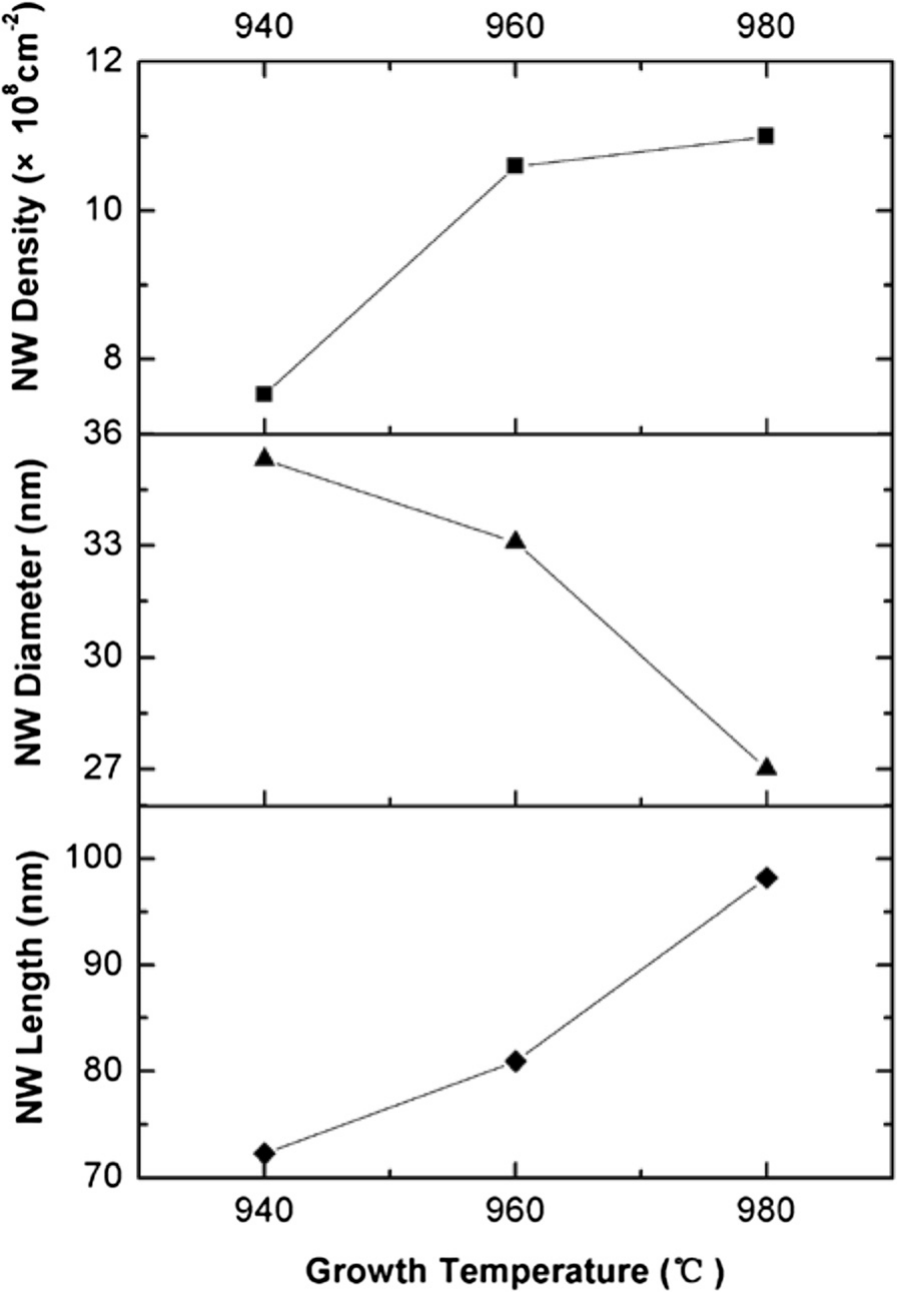
The NW density, average diameter, and length achieved at different growth temperatures. *Solid lines* are guides to the eye

Samples E, C, and F were grown at 940, 960, and 980 °C, respectively, with the same aluminization temperature of 900 °C. In our experiments, AlN NWs cannot be obtained at substrate temperatures below 940 °C, and the temperature exceeding 980 °C is limited by the MBE system. The influence of growth temperature on the characteristics of the AlN NWs is shown in Fig. 4. It can be seen that the density and length of AlN NWs tend to increase while the diameter decreases with increasing growth temperature. As Consonni et al. reported [12], the nucleation islands can thermodynamically transform to NWs on critical condition, and higher growth temperature promotes this transformation. At a certain growth temperature, not all of the nucleation islands can transform to NWs, and an increased growth temperature promotes more nucleation islands developing to NWs, hence the NW density increases. However, limited by the total amount of the nucleation islands obtained under a certain aluminization condition, the NW density tends to saturate as shown in Fig. 4. Higher growth temperature also improves Al migration and then increases the amount of Al adatoms that migrate to the top of NWs; therefore, the axial growth rate of NWs increases [13]. Moreover, the incorporation of adatoms on the NW sidewalls is suppressed at higher growth temperature; thus, the radial growth rate decreases. The similar phenomena were also observed in GaN NW growth [14].

**Fig. 5:**
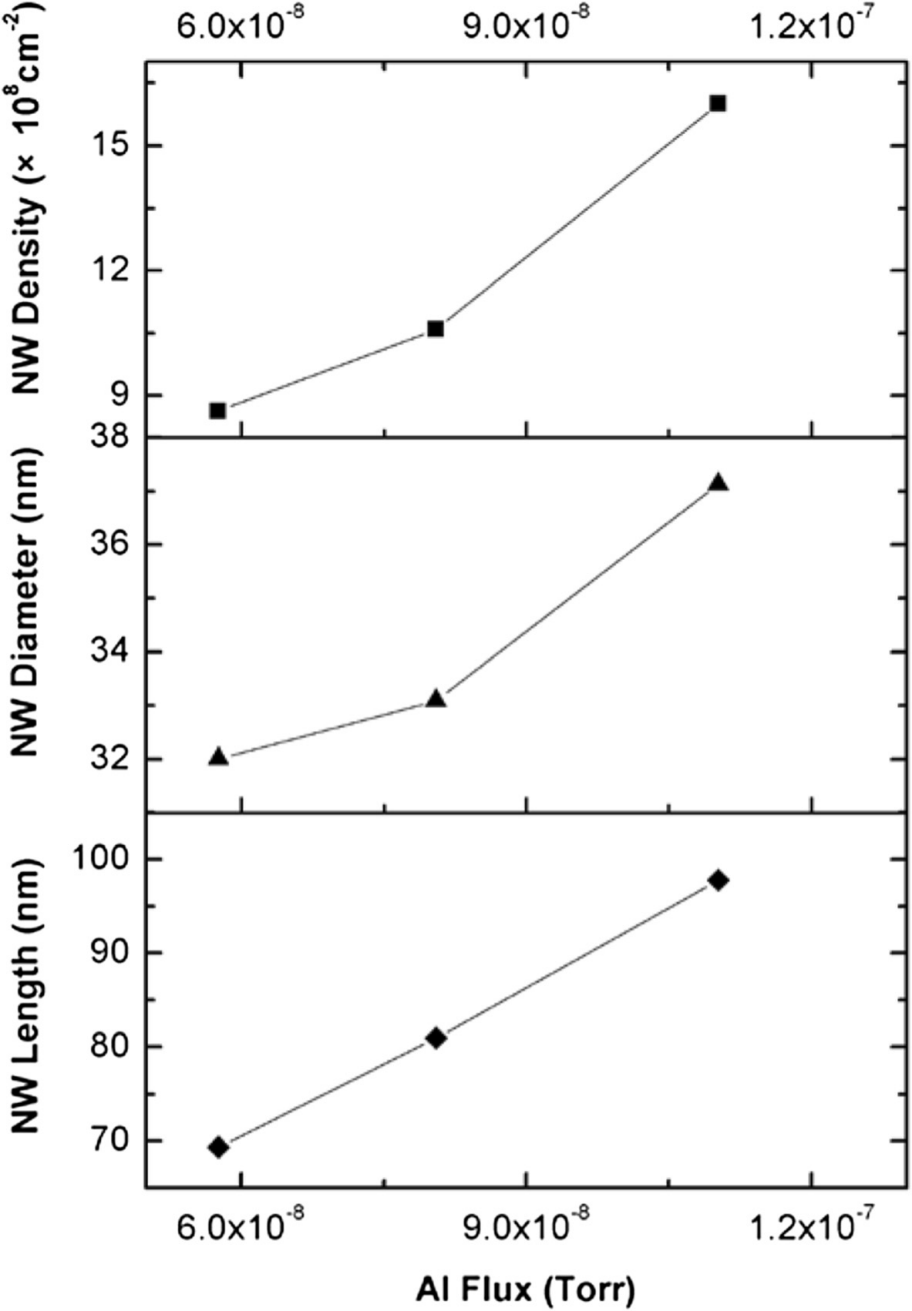
The NW density, average diameter, and length versus Al flux. *Solid lines* are guides to the eye

The influence of Al flux on the morphologies of AlN NWs can be analyzed by comparing samples G, C, and H that were grown using different Al source temperatures while keeping the N_2_ flow rate constant. The corresponding Al fluxes were measured to be 5.8 × 10^−8^, 8.1 × 10^−8^, and 1.1 × 10^−7^ Torr, respectively. Figure 5 shows that the density, average diameter, and length of AlN NWs increase with the Al flux. The increase of NW diameter and length can be understood straightforwardly since increased Al flux will result in more Al atoms incorporating in both the tops and the sidewalls of NWs. Meanwhile, the possible reason for the increase of the NW density is that a higher Al flux will benefit the thermodynamic transformation of nucleation islands to NWs.

**Fig. 6:**
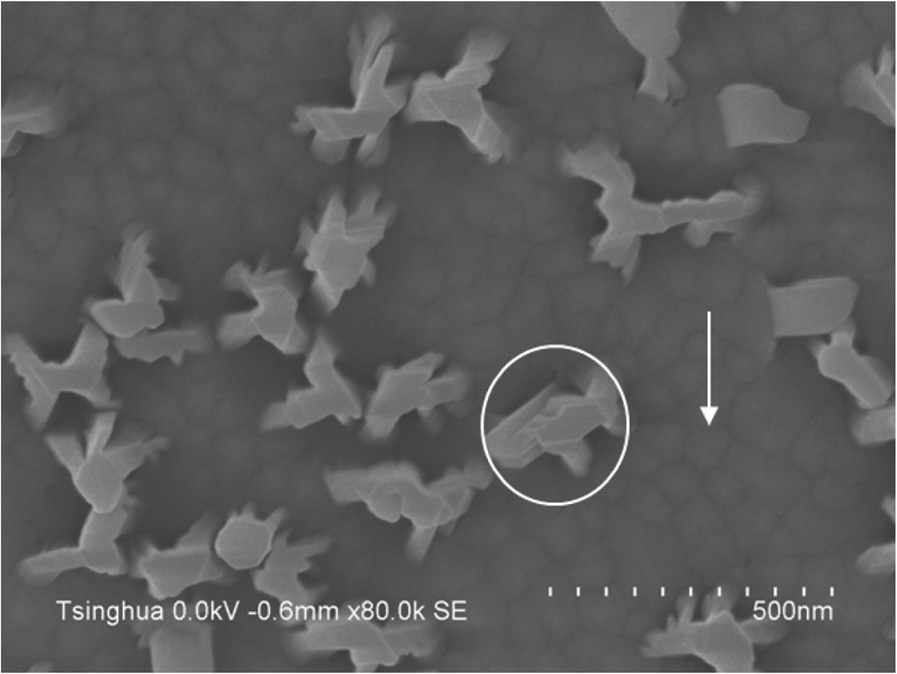
Top-view SEM image of AlN NWs. The *white circle* highlights the coalescence of NWs, and the *white arrow* highlights the island-like film

Samples J and K were grown under the same conditions as samples H and C, respectively, except for longer growth time. Figure 6 shows the typical SEM image of AlN NWs grown for 3 h. The NW density decreases while the NW length and diameter increase compared with the samples grown for 1 h. For example, the NW density, average diameter, and length of sample C are 1.1 × 10^9^ cm^−2^, 33.1 nm, and 80.9 nm, while those of sample K are 7.0 × 10^8^ cm^−2^, 88.2 nm, and 963.3 nm, respectively. Obviously, the axial growth rate is far higher than the radial growth rate. One possible reason for the decrease of NW density is the coalescence of NWs [15], an evidence to which is the NW highlighted by the white circle in Fig. 6. It should also be noted that, as obviously seen in Fig. 6, the AlN NWs are surrounded by island-like AlN films highlighted by the white arrows in the figure. This issue will be discussed below.

**Fig. 7:**
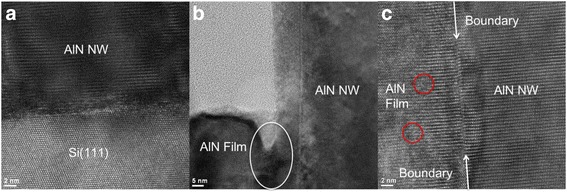
Cross-section HRTEM images of AlN NWs. **a** Interface area between an AlN NW and the Si (111) substrate. **b** Image of an NW and the film connecting to it. **c** Magnified image of the area in the *white circle* in (**b**). The *white arrows* indicate the boundary between the AlN NW and the film, and the nearby distorted lattice in the AlN NW can also be clearly observed. The *red circles* highlight the dislocations in the films

The typical cross-section HRTEM images of AlN NWs are shown in Fig. 7. Fig. 7a shows the interface area between an AlN NW and the Si (111) substrate. The upper part of the AlN NW presents good crystalline quality, and there are quite a few crystal defects around the interface, where the domain coincidence matching between AlN and Si is absent. The defects at the bottom of AlN NWs arise to relax the elastic strain. Therefore, the AlN NWs are directly grown on Si instead of Si_3_N_4_, contrary to the situation of GaN NW growth [16]. This is also an evidence for the aluminizing nucleation during which the β-Si_3_N_4_ layer has completely reacted with the Al adatoms.

In Fig. 7b, the island-like film mentioned above can be observed on the left side of the AlN NW, and a magnified image of the boundary area between the NW and the film is shown in Fig. 7c. The NW exhibits nearly perfect crystalline quality, whereas the film presents some dislocations indicated by the red circles. This can be explained by the different growth mechanisms of the NW and film: the NW relaxes its elastic strain by forming crystal defects at the interface between AlN NW and Si, while the strain in the film is relaxed by forming dislocations. As can be seen in Fig. 7c, there is a clear boundary between the NW and the film, and close to the boundary, there are tiny lattice distortions in the AlN NW extruded by the left-side film. This implies that the island-like films and the NWs grow separately at first and then connect to each other after the films grow laterally. It is elucidated that due to the small Al migration length, the Al adatoms absorbed on the substrate area between NWs cannot totally migrate to the nearby NWs but instead form the island-like films under a relatively high V/III ratio.

**Fig. 8:**
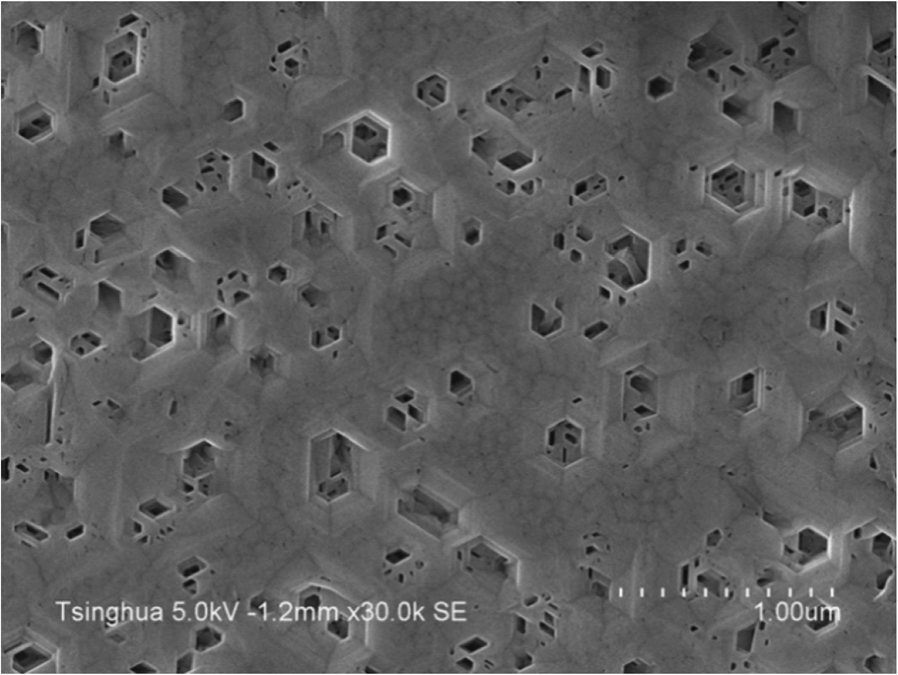
SEM image of the surface of the AlN NW sample etched by 10 % KOH solution for 60 s

The reason for the formation of island-like films instead of newly nucleated NWs may be related to the polarity of AlN crystal. Fernández-Garrido et al. [17] have made a statement that all spontaneously formed nitride NWs by MBE are N-polar, and NWs cannot grow on III-polar nitride epilayers. On the other hand, we have already reported that the AlN islands formed after the aluminization process are N-polar, while the AlN films directly grown on β-Si_3_N_4_ are Al-polar [10]. At the beginning of AlN NW growth, the bare Si (111) surface between AlN islands is unintentionally nitridated to form β-Si_3_N_4_. Then, based on this β-Si_3_N_4_, Al-polar AlN films are formed because the Al adatoms prefer to react with active N radicals rather than β-Si_3_N_4_. In order to examine the polarities of the AlN NWs and island-like films, the sample was treated in 10 % KOH solution for 60 s at 80 °C. KOH solution can only etch N-polar AlN, while Al-polar AlN is resistant to KOH [18]. The SEM image of the etched surface is shown in Fig. 8. It can be seen that there are no NWs remaining on the surface but many hexagonal holes which have nearly the same density and average diameter as the AlN NWs, and the island-like films around the holes are not etched, almost keeping the original morphologies. This is a clear evidence that the AlN NWs are N-polar whereas the films are Al-polar. Furthermore, the residuals in the holes resulted from the anisotropic etching rate with respect to different crystalline planes, and some small holes can be attributed to the N-polar inversion domains in the films.

In order to suppress the growth of the island-like film between NWs, the influence of V/III ratio on the two growth modes was further studied. Table 2 compares the average NW lengths, the island-like film thicknesses, and the wire length/film thickness ratios (W/F ratio) of different samples grown under various V/III ratios. The film thickness was monitored in situ by a reflectivity measurement system, which has been manifested to consist of TEM measurement. Comparing samples J, K, and L, it is found that while keeping N flux constant, both the NW length and the film thickness decrease with Al flux; however, the W/F ratio increases with decreasing Al flux. This means that increasing the V/III ratio suppresses the growth of the AlN film. As for samples K and M, when keeping the Al flux as constant, the NW density, length, and diameter increase with N_2_ flow rate while the film thickness decreases. Therefore, it can be concluded that the growth of the AlN film is suppressed while the growth of AlN NWs is promoted by increasing the V/III ratio. This result is consistent with that shown in Fig. 5, which implies that the NW density, diameter, and length increase with V/III ratio when using the equivalent Al flux. However, in the case of sample O, though the film thickness decreases when the V/III ratio increases further, the NW length and the W/F ratio decrease instead. This may be due to that a significantly high V/III ratio is favorable for the growth of AlN NW sidewall facets, which results in the increased radial growth rate but decreased axial growth rate.

**Table 2:** Influence of V/III ratio on the formation of island-like film

Samples	Average NW length (nm)	Film thickness (nm)	W/F ratio	NW density (cm^−2^)	NW diameter (nm)
J	1046.3	658	1.59	1.4 × 10^9^	107.8
K	963.3	541	1.78	7.0 × 10^8^	88.2
L	437.0	235	1.86	2.4 × 10^8^	56.4
M	990.0	441	2.24	1.6 × 10^9^	95.7
O	734.8	411	1.79	1.9 × 10^9^	118.1

**Fig. 9:**
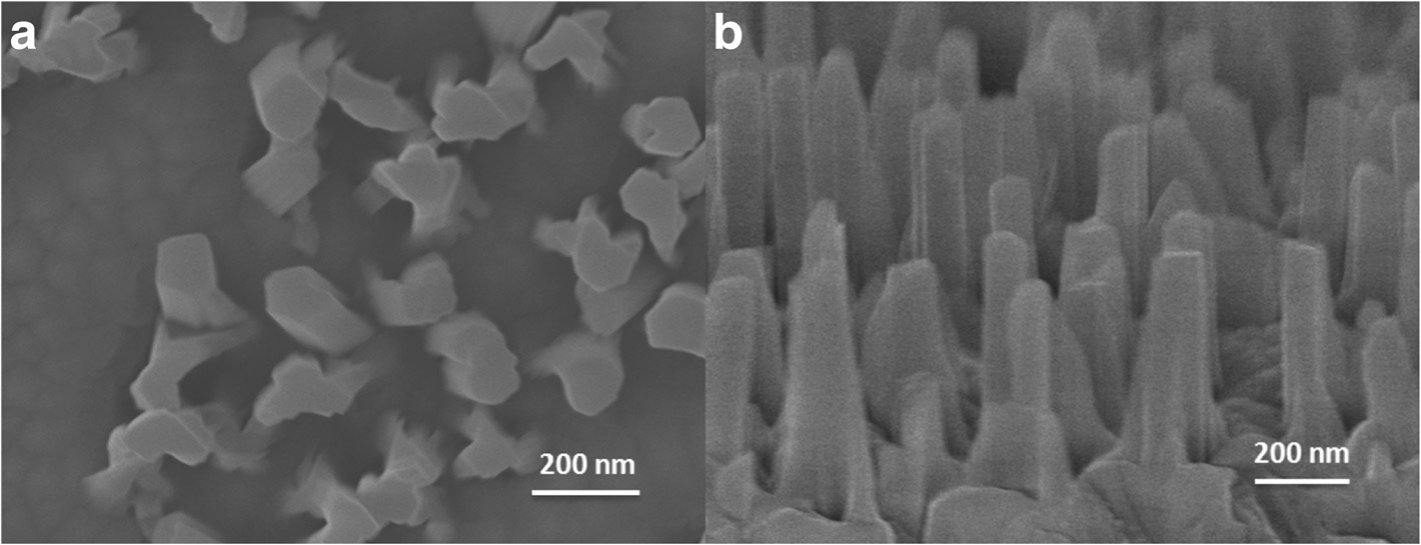
SEM images of AlN NWs. **a** A top-view image of NWs. **b** A bird’s-eye-view image taken with a 70° angle

It can also be observed from the SEM images that, when the V/III ratio changes, the morphologies of the AlN NWs also change. For sample M, the NW density is 1.6 × 10^9^ cm^−2^; however, by taking the criteria established by Fernández-Garrido et al*.* [19], the density of distinguishable single NW is only 1 × 10^8^ cm^−2^, accounting for 6.3 % of the total NWs. Based on the growth conditions of sample M, the Al source temperature was elevated to 1240 °C to grow sample P. The sample presents a NW density of 1.8 × 10^9^ cm^−2^ as well as a significantly increased density of distinguishable single NW of 3.9 × 10^8^ cm^−2^, accounting for 21.7 % of the total NWs. Finally, by increasing the aluminization temperature to 950 °C based on the conditions of sample P, sample Q presents a NW density of 1.9 × 10^9^ cm^−2^ and a density of distinguishable single NW of 6.3 × 10^8^ cm^−2^, accounting for 33.2 % of the total NWs, as shown in Fig. 9.

## Conclusions

In conclusion, an aluminizing nucleation process has been demonstrated to improve the spontaneous growth of AlN NWs by PA-MBE. The AlN NWs are grown from the nuclei formed by the aluminization process; therefore, their morphologies can be controlled by the aluminization parameters. It is found that an elevated growth temperature promotes Al migration and the conversion from nuclei to NWs, while increasing Al flux results in more Al atoms incorporating in both the tops and the sidewalls of NWs as well as a promoted nucleus transformation rate. SEM and TEM measurements reveal that there are island-like films growing between the AlN NWs. Contrary to the N-polar nature of AlN NWs, the films are proved to be Al-polar which can explain the absence of newly formed NWs. Increasing the V/III ratio will promote NW growth while suppressing film growth.

## Abbreviations

AFM: Atom force microscopy
FIB: Focused ion beam
HRTEM: High-resolution transmission electron microscopy
LED: Light-emitting diode
NW: Nanowire
PA-MBE: Plasma-assisted molecular beam epitaxy
RHEED: Reflection high-energy electron diffraction
SEM: Scanning electron microscopy
W/F ratio: Wire length/film thickness ratio

## References

[1] Ma Y, Guo X, Wu X, Dai L, Tong L (2013). Semiconductor nanowire lasers. Adv Opt Photon.

[2] Dong YJ, Tian BZ, Kempa TJ, Lieber CM (2009). Coaxial group III-nitride nanowire photovoltaics. Nano Lett.

[3] Maier K, Helwig A, Müller G, Becker P, Hille P, Schörmann J (2014). Detection of oxidising gases using an optochemical sensor system based on GaN/InGaN nanowires. Sensors Actuators B Chem.

[4] Holmes MJ, Choi K, Kako S, Arita M, Arakawa Y (2014). Room-temperature triggered single photon emission from a III-nitride site-controlled nanowire quantum dot. Nano Lett.

[5] Arafin S, Liu X, Mi Z (2013). Review of recent progress of III-nitride nanowire lasers. J Nanophotonics.

[6] Wang Q, Connie AT, Nguyen HP, Kibria MG, Zhao S, Sharif S (2013). Highly efficient, spectrally pure 340 nm ultraviolet emission from AlxGa(1)-xN nanowire based light emitting diodes. Nanotechnology.

[7] Bourret A, Barski A, Rouviere JL, Renaud G, Barbier A (1998). Growth of aluminum nitride on (111) silicon: microstructure and interface structure. J Appl Phys.

[8] Landré O, Fellmann V, Jaffrennou P, Bougerol C, Renevier H, Cros A (2010). Molecular beam epitaxy growth and optical properties of AlN nanowires. Appl Phys Lett.

[9] Wang Q, Zhao S, Connie AT, Shih I, Mi Z, Gonzalez T (2014). Optical properties of strain-free AlN nanowires grown by molecular beam epitaxy on Si substrates. Appl Phys Lett.

[10] Hu J, Hao Z, Niu L, Yanxiong E, Wang L, Luo Y (2013). Atomically smooth and homogeneously N-polar AlN film grown on silicon by alumination of Si_3_N_4_. Appl Phys Lett.

[11] Ahn H, Wu CL, Gwo S, Wei CM, Chou YC (2001). Structure determination of the Si_3_N_4_/Si(111)-(8 × 8) surface: a combined study of Kikuchi electron holography, scanning tunneling microscopy, and ab initio calculations. Phys Rev Lett.

[12] Consonni V, Hanke M, Knelangen M, Geelhaar L, Trampert A, Riechert H. Nucleation mechanisms of self-induced GaN nanowires grown on an amorphous interlayer. Phys Rev B. 2011;83(3). doi:10.1103/PhysRevB.83.035310.

[13] Consonni V, Dubrovskii VG, Trampert A, Geelhaar L, Riechert H. Quantitative description for the growth rate of self-induced GaN nanowires. Phys Rev B. 2012;85(15). doi:10.1103/PhysRevB.85.155313.

[14] Fernández-Garrido S, Grandal J, Calleja E, Sánchez-García MA, López-Romero D (2009). A growth diagram for plasma-assisted molecular beam epitaxy of GaN nanocolumns on Si (111). J Appl Phys.

[15] Consonni V, Knelangen M, Trampert A, Geelhaar L, Riechert H (2011). Nucleation and coalescence effects on the density of self-induced GaN nanowires grown by molecular beam epitaxy. Appl Phys Lett.

[16] Stoica T, Sutter E, Meijers RJ, Debnath RK, Calarco R, Luth H (2008). Interface and wetting layer effect on the catalyst-free nucleation and growth of GaN nanowires. Small.

[17] Fernandez-Garrido S, Kong X, Gotschke T, Calarco R, Geelhaar L, Trampert A (2012). Spontaneous nucleation and growth of GaN nanowires: the fundamental role of crystal polarity. Nano Lett.

[18] Zhuang D, Edgar JH (2005). Wet etching of GaN, AlN, and SiC: a review. Mater Sci Eng R Rep.

[19] Brandt O, Fernández-Garrido S, Zettler JK, Luna E, Jahn U, Chèze C (2014). Statistical analysis of the shape of one-dimensional nanostructures: determining the coalescence degree of spontaneously formed GaN nanowires. Cryst Growth Des.

